# Clinical and Radiographic Outcomes of ESIN, Plate, and K-Wire Fixation in Pediatric Tibial DDMJ Fractures: A Retrospective Comparative Study

**DOI:** 10.3390/children12101345

**Published:** 2025-10-07

**Authors:** Nevzat Gönder, Çağrı Karabulut, Musa Alperen Bilgin, İbrahim Halil Demir, Ramazan Parıldar, Beytullah Unat, İbrahim Halil Rızvanoğlu

**Affiliations:** 1 Department of Orthopedics and Traumatology, Faculty of Medicine, Gaziantep University, 27000 Gaziantep, Türkiye; 2 Department of Orthopedics and Traumatology, T.C. Ministry of Health Pazarcık State Hospital, 46700 Kahramanmaraş, Türkiye; cagri.karabulut@saglik.gov.tr; 3 Department of Orthopedics and Traumatology, T.C. Ministry of Health Çorum Iskilip State Hospital, 19400 Çorum, Türkiye; musaalperen.bilgin@saglik.gov.tr; 4 Department of Orthopedics and Traumatology, Gaziantep City Hospital, 27470 Gaziantep, Türkiye; ibrahimhalil.demir@saglik.gov.tr; 5 Department of Orthopaedics and Traumatology, T.C. Ministry of Health 25 Aralık State Hospital, 27090 Gaziantep, Türkiye; ramazan.parildar@saglik.gov.tr; 6 Department of Orthopedics and Traumatology, Faculty of Medicine, Gaziantep Islam Science and Technology University, 27010 Gaziantep, Türkiye; beytullah.unat@gibtu.edu.tr; 7 Department of Orthopedics and Traumatology, NCR International Hospital, 27090 Gaziantep, Türkiye; ibrahimhalil.rizvanoglu@saglik.gov.tr

**Keywords:** distal tibia, diaphysis, metaphysis, pediatric, plate, K-wire, elastic stable intramedullary nail

## Abstract

**Background:** Tibial distal diaphyseal–metaphyseal junction (DDMJ) fractures are rare in children and pose therapeutic challenges due to their morphology and risk of displacement. This study compared the clinical, radiological, and economic outcomes of elastic stable intramedullary nailing (ESIN), plate fixation, and Kirschner wire (K-wire) fixation. **Methods:** A retrospective review was conducted on 64 patients (6–15 years) treated between 2014 and 2023. Patients were grouped according to fixation method. Demographic, operative, radiographic, functional (AOFAS), complication, and cost data were analyzed. **Results:** The K-wire group, plate group, and ESIN group consisted of 27, 19, and 18 patients, respectively. The mean follow-up duration was 18.03 ± 6.87 months. Of the patients, 38 were male and 26 were female. Concomitant fibula fractures were present in 43 patients and were not present in 21 patients. AOFAS scores were highest in the plate group at the 4th month, while they were similar in all groups in the subsequent follow-ups. The costliest method was plate (2517.64 ± 104.83 $) (*p* = 0.001). **Conclusions:** All three fixation methods provided satisfactory long-term outcomes. Plate fixation offers faster early recovery but at higher cost and risk of soft-tissue complications; ESIN balances stability and invasiveness; K-wire is economical but less stable. Treatment choice should be individualized according to fracture pattern, patient factors, and resource availability.

## 1. Introduction

In the pediatric population, tibia fractures are the third most common fractures, following forearm and femur fractures, and are the second most frequent type of fracture requiring hospitalization [1,2,3,4]. The incidence of tibia fractures among all pediatric fractures is 15%, while distal metaphyseal fractures occur at a highly rare rate of 0.35% to 0.45% [1,2,3,4]. Although the treatment of pediatric tibia fractures varies depending on many factors such as the age of the patient, localization of the fracture, accompanying injuries, and general condition of the patient, it is mostly treated conservatively if it is within acceptable reduction limits [5]. Acceptable reduction limits are less than 10 degrees of varus, less than 8 degrees of valgus, up to 12 degrees of procurvatum, and up to 6 degrees of recurvatum [5]. The tibia is most susceptible to trauma at the transition from the midshaft to the distal third due to the change in the bone’s cross-sectional shape from triangular to round [6]. Fractures at the distal diaphyseal–metaphyseal junction (DDMJ) of the pediatric tibia pose a significant challenge in orthopedic surgery due to the transitional anatomical and biomechanical properties. Although the primary treatment for fractures in this location is conservative, in cases with severe displacement, inability to reduce, instability, associated open fractures, neurovascular injury, and polytrauma, additional surgical methods are often required to prevent displacement and maintain reduction [5,7,8]. Among the various surgical options, elastic stable intramedullary nailing (ESIN), plate fixation, external fixation, and Kirschner wire (K-wire) fixation are the most commonly used approaches.

ESIN has gained widespread acceptance thanks to its minimally invasive nature, benefits of early mobilization, and particularly excellent functional outcomes in diaphyseal fractures [8,9,10]. However, its application in distal metaphyseal regions remains technically challenging due to the short distal fragment and proximity to the physis. Plate fixation provides rigid anatomical stabilization at the cost of extensive soft tissue dissection, while K-wire fixation remains biomechanically weaker in controlling translation and angulation [11,12,13]. In addition to clinical and radiographic outcomes, we analyzed the economic aspects of treatment, as implant selection has significant implications for healthcare systems. While functional recovery may converge over time, differences in implant cost, operative duration, and hospital stay represent a relevant burden for both families and healthcare providers.

Although there are various recommendations for the treatment of this uncommon and problematic fracture in the literature, the literature is limited, and there is no established gold standard treatment approach. In addition, studies have generally focused on the clinical benefits of a single method, and comparative studies are sparse in the literature. To the best of our knowledge, there is no study in the literature that comprehensively compares the three approaches used in our study on pediatric tibial DDMJ fractures.

The objective of this study was to compare the clinical and radiological outcomes of ESIN, plate, and K-wire fixation in pediatric unstable tibial DDMJ fractures; it focused on postoperative alignment, functional recovery based on the American Orthopedic Foot and Ankle Society (AOFAS) score, complication rates, and treatment costs.

## 2. Materials and Methods

After obtaining approval from the ethics committee, skeletally immature patients who were followed up and treated at our hospital for tibial DDMJ fractures, with or without an accompanying fibula fracture, were included in this study. The medical records of 64 patients who met the inclusion criteria and were treated between September 2014 and January 2023 were retrospectively reviewed. An initial reduction attempt was performed in all patients for conservative treatment. Unstable fractures that could not be reduced or that were not within acceptable reduction limits were evaluated.

**Figure 1:**
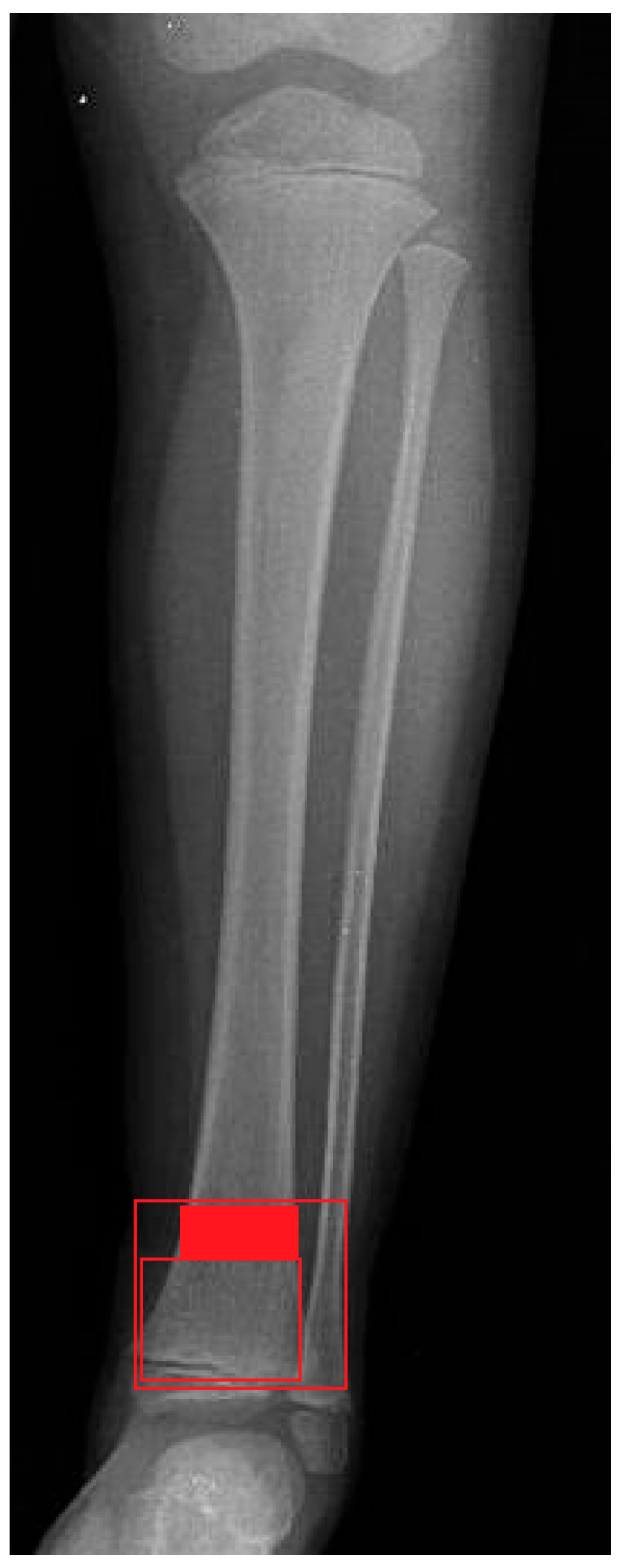
X-ray depiction of the DDMJ fracture.

DDMJ is delineated according to “The AO Pediatric Comprehensive Classification of Long Bone Fractures,” wherein the metaphyseal region is represented by a rectangle encompassing the base of the growth plate of both the distal tibia and fibula (the large square at the distal tibia and fibula). Additionally, a smaller square is positioned adjacent to the tibial epiphysis, with a height equivalent to the broadest section of the tibial epiphysis. The region of the distal tibia located between two squares is designated as the DDMJ [14,15] (Figure 1).

### 2.1. Patients’ Demographics

Age, gender, affected side, trauma mechanism, fracture type, accompanying fibula fracture, additional surgical procedures, duration of surgery, duration of union, complications, and total costs during treatment and follow-up were obtained through file review. Inclusion criteria:

∗ Patients aged 6–16 years.
∗ Diagnosis of unstable tibial DDMJ fracture.
∗ Treated with one of three fixation methods: K-wire, ESIN, or plate osteosynthesis.
∗ Closed fractures.
∗ Minimum 12 months of follow-up with complete clinical and radiographic records available.

Exclusion criteria:

∗ Pathological fractures.
∗ Polytrauma patients or those with metabolic bone disease.
∗ Incomplete records or follow-up <12 months.
∗ Open fractures.

### 2.2. Radiographic Assessment

Anteroposterior and lateral radiographs of the leg and ankle were obtained for all patients on the injured side. Fracture types were classified as transverse, oblique, or comminuted based on radiographic assessment. Anteroposterior radiographs were used to assess varus or valgus deformities based on the displacement of the distal fragment relative to the tibial shaft axis. Lateral radiographs were used to assess the degree of recurvatum or procurvatum angulation.

**Figure 2:**
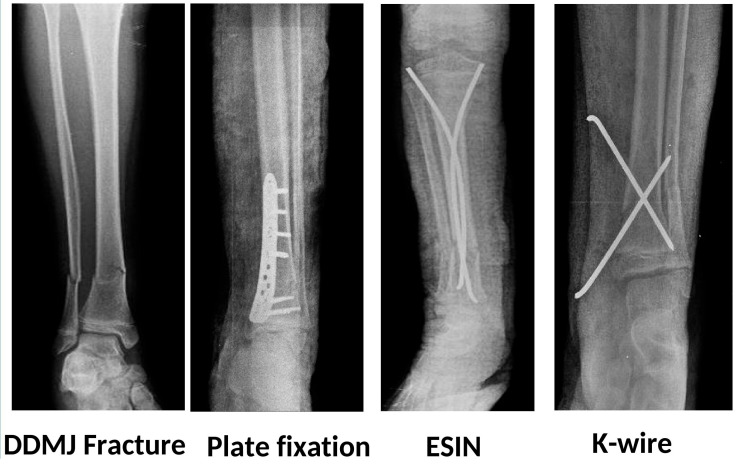
Radiographic images of patients who undergone plate fixation, ESIN, and K-wire fixation, respectively.

Patients were classified based on the fixation method: K-wire, plate, and ESIN (Figure 2). In the group treated with K-wire, if a fibular fracture was present, the fibula was fixed with a single retrograde K-wire. In the other two groups, no fixation was performed even if a fibula fracture was present. In the plate group, no extensive surgical dissection was performed, and minimal invasive plating techniques were followed. In the TEN group, fixation was achieved through antegrade insertion of two elastic nails, ranging in size from 2 to 4 mm depending on the morphology of the tibia.

### 2.3. Follow-Up

In the postoperative period, if no additional complications were present, patients were discharged on the 3rd day on average. After surgery, all patients were stabilized with a short leg splint for 3 weeks. Immediately after the splint was removed, both active and passive ankle movements were initiated. In the 1st month, weight-bearing was gradually allowed based on the union status observed in radiographs.

During follow-up, radiographs were evaluated by a senior orthopedist; however, they were not included in the care of the patients. Measurements were conducted on two occasions, one week apart. All angular and translation measurements were made manually on standardized AP and lateral films and ICC values were added. All cases were evaluated with full-leg AP and lateral radiographs. In addition, after fracture healing, a standing full-leg length radiograph was used to assess lower extremity alignment and to evaluate any length discrepancy in the extremities. Length differences under 1 cm were followed up as they did not cause a clinical problem. Union was defined as healing of at least 3 out of 4 cortices on anteroposterior and lateral radiographs. Varus, valgus, recurvatum, and procurvatum values were measured through the radiographs of the patients at the fourth- and eighth-month examinations and at the last follow-up.

Functional recovery was evaluated at the fourth, eighth, and final clinical examinations. The Ankle-Hindfoot Scale, which was devised by the American Orthopedic Foot and Ankle Society (AOFAS), was employed to evaluate the clinical outcomes.

Economic evaluation was performed from the hospital/provider perspective, covering direct medical costs from surgery through follow-up. Costs included implants and disposables, operating room time, length of hospital stay, outpatient visits, radiographs, and implant removal when performed. Complication-related treatments were also incorporated. Unit costs were obtained from the hospital’s official tariff list and standardized to the same price year. For international comparability, values were also expressed in USD equivalents. Indirect costs (transportation, family burden, productivity loss) were not included. There is no standard decision-making guide for choosing between plate, ESIN, and K-wire fixation. But practical rule of thumb: plate: best for early function, ESIN: balanced choice, K-wire: most economical, less stable.

### 2.4. Statistical Analysis

The commitment of numerical variables to a normal distribution was evaluated using the Shapiro-Wilk test. The ANOVA test was employed to compare regularly distributed variables across three groups, while the Kruskal-Wallis and Dunn tests were utilized for non-normally distributed variables among the same groups. Two-way repeated measures ANOVA and LSD tests were employed to assess the variation in groups over time and the interaction of group measurements. The relationships among categorical variables were analyzed using the Chi-square test. The intraclass correlation coefficient (ICC) was employed to determine the repeatability of measurements. The analyses were conducted using SPSS version 22.0 (IBM SPSS Corp.; Armonk, NY, USA) for Windows. A *p*-value of less than 0.05 was deemed significant.

## 3. Results

The K-wire group, plate group, and ESIN group consisted of 27, 19, and 18 patients, respectively. The patients’ mean age was 10.23 ± 2.08 years. Although the mean age in the plate group was slightly higher, it was not statistically significant (*p* = 0.11). The mean follow-up duration was 18.03 ± 6.87 months. Of the patients, 38 were male and 26 were female. In 35 patients, the fracture occurred on the right side, while in 29 patients, it was on the left side. Regarding fracture type, 32 patients had transverse fractures, 27 had oblique fractures, and 5 had comminuted fractures. The most common injury mechanism was a motor vehicle accident occurring in 17 patients, followed by riding a bike in 13, electric scooter accidents in 12, sports-related injuries in 12, and falls from height in 10 patients. Concomitant fibula fractures were present in 43 patients and were not present in 21 patients. No statistically significant difference was observed among the groups regarding demographic data (Table 1). The mean operation time was 76 ± 22 min in the plate group, 56 ± 18 in the ESIN group, and 50 ± 20 in the K-wire group. The operation time in the plate group was significantly longer compared to both ESIN and K-wire groups (*p* = 0.001).

When the radiological data, including varus-valgus, procurvatum, recurvatum, and sagittal-coronal translation, were compared among the groups before, after, and at the last follow-up, no statistically significant difference was found (Table 2a,b). The ICC values for angulations in the coronal plane were preoperative (0.97), postoperative (0.93), and final follow-up (0.87), respectively. The ICC values for angulations in the sagittal plane were preoperative (0.98), postoperative (0.84), and final follow-up (0.82), respectively. The ICC values for translations in the sagittal plane were preoperative (0.94), postoperative (0.86), and final follow-up (0.85), respectively. The ICC values for translations in the coronal plane were preoperative (0.98), postoperative (0.84), and final follow-up (0.97), respectively. The intraclass correlations were perfect and almost perfect for all measurements. The mean union time was found to be 7.92 ± 1.25 weeks in the plate group, 8.22 ± 1.64 in the ESIN group, and 8.2 ± 1.56 in the K-wire group, and there was no statistically significant difference among the groups (*p* = 0.741).

**Figure 3:**
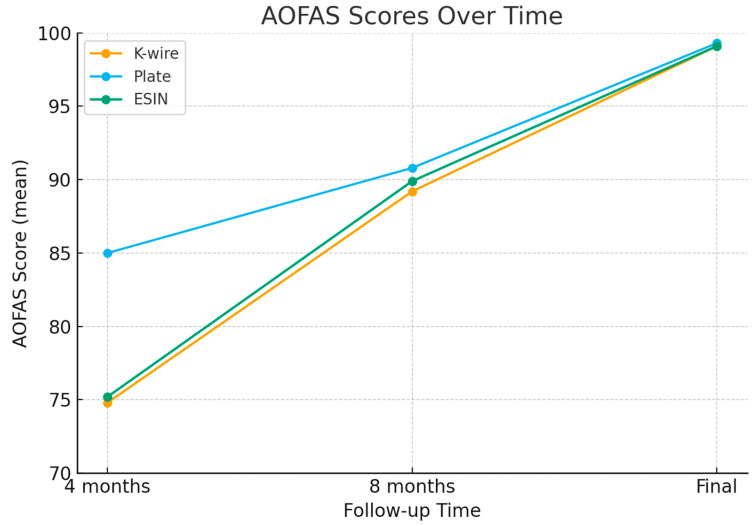
Temporal variation in AOFAS scores by cohort.

In terms of clinical evaluation, clinical scores in the plate group were significantly higher compared to the ESIN and K-wire groups at the 4th postoperative month. However, at the 8th month and final follow-up, there were no statistically significant differences in clinical outcomes among the groups (Table 3). In all groups, clinical scores improved significantly from the 4th to the 8th month and from the 8th month to the final follow-up (Figure 3).

**Figure 4:**
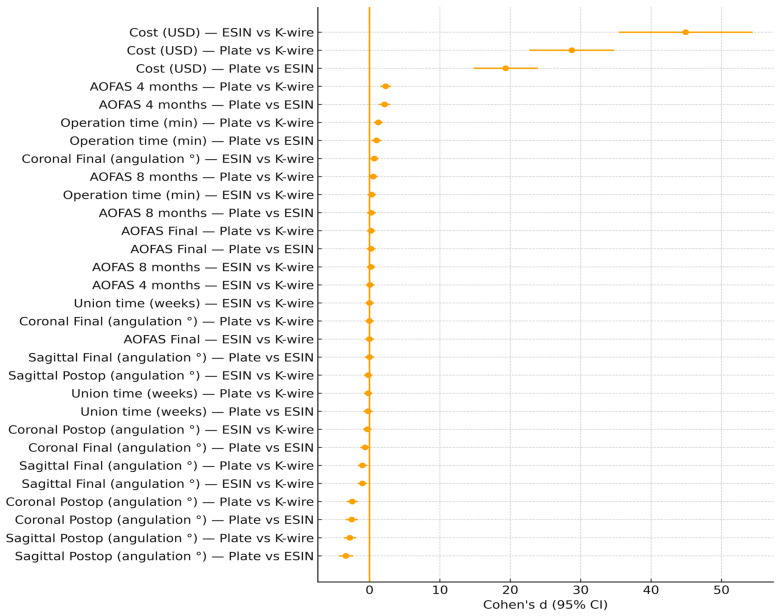
Cohen effect sizes by outcome and comparison.

Our effect size analysis provides additional insight into the clinical relevance of the findings (Figure 4). Union time demonstrated negligible differences across fixation methods (Cohen’s d ≤ 0.2), confirming that healing potential in pediatric patients is independent of implant choice. In contrast, operation time revealed large effects, with plate fixation requiring approximately 20–26 min longer than ESIN or K-wire. This difference is not only statistically significant but also clinically relevant in the context of anesthesia exposure and operating room efficiency. Cost analysis further highlighted large absolute mean differences, with plate fixation being nearly twice as expensive as ESIN and more than three times as costly as K-wire, underscoring the economic implications of implant choice. Functional outcomes measured by AOFAS showed a moderate effect size favoring plates at 4 months, reflecting faster early recovery, but these advantages disappeared by final follow-up as remodeling and rehabilitation equalized outcomes across groups. Radiographic outcomes similarly demonstrated large postoperative effect sizes favoring plate fixation for coronal and sagittal alignment, yet these differences became trivial at consolidation, confirming the strong remodeling capacity of the pediatric tibia. Taken together, these findings suggest that while plates may provide superior early alignment and functional recovery, their higher operative burden and cost must be weighed against the equally effective long-term outcomes of ESIN and K-wire fixation.

In the postoperative period, one patient in the K-wire group experienced loss of reduction and was re-reduced and fixed with K-wires in the operating room. In the K-wire group, three patients developed pin site infections; however, they healed uneventfully with daily dressing changes and antibiotherapy. In the plate group, one patient developed necrosis around the incision site, which required debridement followed by closure with a fasciocutaneous flap. In the plate group, two patients developed superficial soft tissue infections, but they healed uneventfully with antibiotherapy. In the ESIN group, one patient developed skin irritation at the TEN entry point, and two patients developed superficial soft tissue infections; however, all recovered without complications. No patient developed clinically or radiologically significant overgrowth or limb length discrepancy. At the final follow-up, no patient exhibited limp, and bilateral lower extremity movements were symmetrical. No patient developed physeal arrest or angular deformity related to it. Implant removal was performed in the 10th week in the K-wire group, in the 6th month in the ESIN group, and in the 8th month in the plate group.

**Figure 5:**
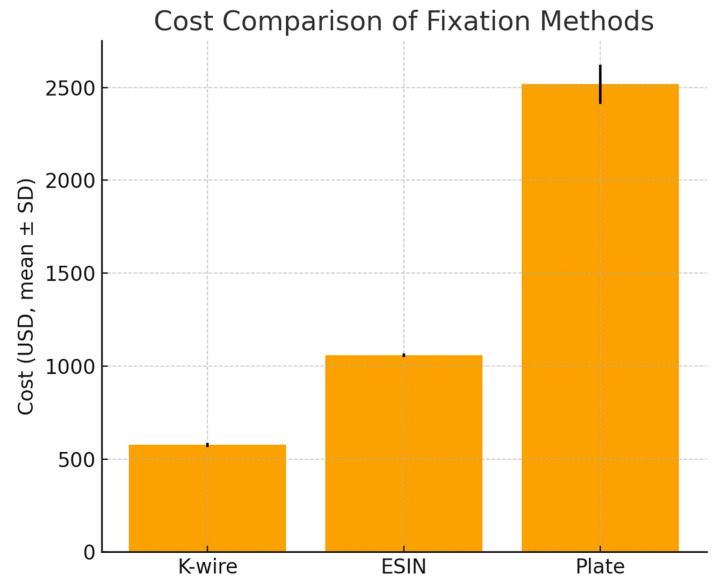
Total cost comparison between the groups.

Considering all costs during the treatment process, the cost analysis revealed that the most cost-effective method was K-wire (576 ± 11.06 $), ESIN (1056.95 ± 10.14 $) was more expensive compared to K-wire. The costliest method was in the plate (2517.64 ± 104.83 $) group (*p* < 0.001). Statistically, the costliest group was also the plate group (Figure 5).

## 4. Discussion

The majority of distal tibial metaphyseal fractures in children recover uneventfully with closed reduction and casting, without the need for surgical intervention. Treatment should be planned considering the complications of casting, such as compartment syndrome, joint stiffness, muscle atrophy, and loss of reduction [16]. In recent years, the rate of surgical intervention for pediatric fractures, not only tibial fractures in children but also fractures in general, has been increasing worldwide [8,13,17,18]. Surgical intervention becomes inevitable in fractures where closed reduction and casting fail to achieve or maintain acceptable alignment, rotation, and length. Conservative treatment of severely displaced tibial DDMJ fractures is more challenging and often requires intervention [8,13,18].

When the literature on surgical approaches for tibial DDMJ fractures is reviewed, there is no clear surgical option or standardized algorithmic approach. ESIN, plate osteosynthesis, external fixation, K-wire fixation, and intrafocal pinning are the most commonly used surgical approaches [18,19,20,21,22,23,24]. These methods have their own advantages and disadvantages. For example, while ESIN offers advantages such as minimal invasiveness, low complication rates, and early physiotherapy, it may lead to inadequate stabilization and malreduction in tibial DDMJ fractures due to insufficient filling of the medullary cavity [2,18,22]. Although plate osteosynthesis provides rigid fixation and allows for early mobilization, potential issues such as soft tissue complications, bleeding, delayed union, and the need for implant removal should not be overlooked [25]. Although studies report that external fixation has minimal soft tissue damage, is easy to apply, and has low complication rates, there are also studies highlighting its disadvantages, such as long union time, poor functional outcomes, excessive scarring, difficulty with daily activities, and pin site infections [8,18,24]. K-wire osteosynthesis can be used as the first choice in simple transverse or short oblique fractures [25]. The advantages of percutaneous osteosynthesis with K-wires include less soft tissue damage, lower cost, and availability under all conditions, while the disadvantages include insufficient stabilization, pin migration, loss of reduction, and pin site infections [11,21]. The most significant finding of this study is that while plate fixation provides the best early clinical results, all three fixation methods achieved similar long-term clinical and radiographic outcomes, with K-wire being the most cost-effective approach.

The mean age of the patients included in our study was 10.23 years, which was relatively higher in the plate group, but there was no statistically significant difference among the groups, and this was similar to the studies in the literature [8,21]. Consistent with the proportions in the fracture population of this age group, 59.4% of the patients were male [26]. The most common fracture type was transverse, followed by oblique fractures. Transverse and oblique fractures are the most commonly seen fractures in this area [24]. When the current literature is reviewed, it is observed that there is no standard approach when a fibula fracture accompanies a tibia fracture. However, in cases of bilateral bone fractures, the need for surgical intervention increases as it becomes difficult to maintain stabilization with casting [27,28,29]. In certain studies, fixation is recommended to prevent secondary displacement [30,31]. In our study, 43 patients had concomitant fibula fractures. In our study, among the patients who underwent osteosynthesis with K-wire, 16 patients with a fibula fracture were fixed to prevent secondary displacement and enhance stabilization. However, no difference was observed between the patients who were fixed and those who were not in terms of complication rates, clinical, and radiological outcomes. In their study comparing different pin configurations in distal tibia fractures, Brantley et al. demonstrated that there was no difference between the K-wire configuration and biomechanical properties [11]. In our study, cross fixation with two K-wires was applied in the patients who underwent fixation with a K-wire.

Although there is limited information regarding the surgical duration, it is known that patients undergoing osteosynthesis with ESIN and K-wire have a relatively shorter surgical time compared to the plate group [8,10,29,32]. In our study, the duration of surgery was longer in the plate group, similar to the literature. The duration of union in pediatric distal tibia fractures can vary between 5–40 weeks [32,33,34,35]. While there are studies showing that delayed union occurs in patients treated with plate fixation, Ghannam et al. argued that there was faster union compared to ESIN [23]. In our study, the mean union time was approximately 8 weeks, and there was no significant difference among the groups.

It is a controversial topic how much plaster or splint should be used to stabilize tibial DDMJ fractures in the postoperative period. In patients treated with plates, studies suggesting that stabilization is not necessary are more prevalent, but there are also studies that recommend its early use [13,20]. Although there are studies suggesting that postoperative stabilization without a cast does not lead to any reduction loss in patients treated with ESIN, it continues to be generally used to prevent secondary displacement [8,10,35]. While the principle of ESIN is cast-free follow-up, the fractures in our study were located near the distal metaphyseal level, and we followed this approach due to the worry about loss of stabilization. Our decision for 3-week splinting was based on institutional protocol and concerns about compliance in younger children. Furthermore, in the most recent literature, casting continues to be used even when adequate stabilization of the tibial fracture is achieved with ESIN [36]. In general, the use of a cast after K-wire osteosynthesis is recommended in the literature as well [12]. All of our patients were stabilized with a splint postoperatively, and a circular cast was not used. After the splint was removed, active and passive ankle exercises were started immediately. The 3-week splinting duration in our study is consistent with similar studies in the literature. It is possible that the duration could be shortened, allowing for earlier joint range of motion exercises. However, joint stiffness due to arthrofibrosis is not typically a common complication in the pediatric age group. It is possible to follow up the patients without using a splint; however, in cases where the patient fails to adhere to recommendations and places weight on the limb, reduction loss, implant failure, and the need for re-surgery may occur. To avoid these complications, early stabilization with a splint was preferred for our patients.

One patient in the K-wire group experienced loss of reduction and was re-reduced and fixed with K-wires in the operating room. In the K-wire group, three patients developed pin site infections; however, they healed uneventfully with daily dressing changes and antibiotherapy. In pediatric tibia fractures, complications such as soft tissue infections, osteolysis in the medial fibular cortex, and growth arrest may occur following fixation with a plate [10,13,33]. In our study, in the plate group, one patient experienced necrosis around the incision site, which required debridement followed by closure with a fasciocutaneous flap. Additionally, two patients had superficial soft tissue infections, but these were successfully treated with antibiotherapy without complications. In their study where they applied ESIN to pediatric tibial fractures and published the 20-year results, Pogorelić et al. reported minor complications by 11.36% [10]. These complications included skin blisters, ESIN protrusion, skin irritation at the pin entry point, and a refracture in one patient [10]. In our study, one patient developed skin irritation at the ESIN entry point, and two patients developed superficial soft tissue infections; however, all recovered without complications. No overgrowth or limb length discrepancy due to shortening that would cause clinical and radiological problems occurred in any of the patients, and no refracture developed. In the last examinations, no patient developed physeal arrest or angular deformity related to it. In general, when all complications were considered, there was no statistically significant difference among the groups.

In patients treated with ESIN, to achieve better alignment and stabilization, the divergent intramedullary nailing technique described by Harly et al. can be used instead of the classic ESIN technique [22]. In our study, both sagittal and coronal plane deformities were statistically lower in all groups in the postoperative and final examinations compared to the preoperative period. There was no statistically significant difference among the groups in terms of varus, valgus, recurvatum, procurvatum, and translational values in both the sagittal and coronal planes during the early postoperative period and the final follow-up.

The diminishing effect sizes for residual angulation and translation at final follow-up highlight the well-documented remodeling potential of the pediatric tibia. Remodeling capacity is strongly age-dependent: children under 10–12 years can reliably correct coronal and sagittal plane deformities, whereas adolescents remodel less predictably [37]. In the coronal plane, varus deformities remodel more consistently than valgus deformities, the latter being less responsive and more likely to persist [37,38]. Similarly, in the sagittal plane, flexion deformities remodel more reliably than recurvatum deformities [38]. By contrast, rotational malalignments show little or no spontaneous correction and should always be avoided intraoperatively [39]. Clinically, this supports our observation that although plate fixation demonstrated large postoperative effect sizes for alignment, these differences became negligible at consolidation. In younger patients, even moderate postoperative deviations may be expected to remodel, whereas in older adolescents, achieving near-anatomic reduction remains essential. Surgeons must therefore weigh the potential for natural correction against the invasiveness, cost, and operative time of each fixation method.

In the literature, it has been emphasized that plate osteosynthesis provides better angular stability, making it a suitable option, since it offers the possibility of earlier mobilization and better functional outcomes [13]. The ESIN method is a well-known method with specific indications, and satisfactory clinical results can be achieved when performed in accordance with the rules [34,35]. Although K-wire fixation is less stable compared to other methods, it can provide good clinical results with minimal soft tissue damage and low complication rates [11,20,21]. In our study, AOFAS scores were significantly better in the first 4-month follow-up in the plate group compared to the other groups. There was no statistically significant difference in the AOFAS scores among the groups at the 8th month and final follow-up. In a study conducted by Thabet et al. on skeletally immature patients, no significant difference was found between ESIN, interlocking nail, plate, external fixation, and casting in terms of recovery and complications [32].

When the cost analysis was examined, the group treated with plate osteosynthesis was found to be significantly more expensive than the ESIN and K-wire groups. In the ESIN group, it was more costly compared to the K-wire group. When the literature is examined, the economic cost of osteosynthesis with a plate is high due to both the cost of the implant and the need for implant removal [40].

Our study had certain limitations. Firstly, it was a retrospective study. Secondly, radiation exposure may vary based on the method used, but radiation dosage was not followed in our study. Finally, only angular deformities were evaluated, while rotational deformities were not measured. Measurement error related to X-ray projection variability is also a limitation. Our follow-up imaging protocol was indeed more intensive than required. Two-plane intraoperative radiographs, one at consolidation, and one before implant removal (with an additional early check for K-wires), are sufficient according to published guidelines. While this allowed detailed monitoring, it also increased radiation exposure, which is a methodological limitation. Future studies should adopt standardized protocols that balance patient safety with the principle of minimizing unnecessary imaging. Although we reported the distribution of fracture types (transverse, oblique, comminuted), subgroup analysis was not performed owing to a limited sample size. Cost variability across healthcare systems is another limitation of our study. Future prospective studies ought to categorize outcomes based on fracture morphology, given that stability and remodeling capacity vary among different patterns. The strengths of our study are that, thanks to being a trauma clinic, the number of patients is considered to be sufficient, even for an uncommon fracture.

## 5. Conclusions

Unstable pediatric tibial DDMJ fractures can be successfully treated with plate fixation, ESIN, or K-wires, each offering comparable long-term outcomes. Plate fixation provides faster early recovery but is costly and carries greater soft tissue risk. ESIN offers a balanced option with good stability and moderate cost, while K-wires remain the most economical but less rigid. Surgical choice should therefore be individualized according to fracture pattern, patient needs, and healthcare resources.

## Figures and Tables

**Table 1:** Comparison of demographic data between groups.

Variable	K-Wire (n = 27)	Plate (n = 19)	ESIN (n = 18)	*p*-Value
Sex (M/F)	15/12 (55.6/44.4%)	12/7 (63.2/36.8%)	11/7 (61.1/38.9%)	0.861
Age (years, mean ± SD)	9.78 ± 2.39	11.05 ± 1.47	10.06 ± 1.95	0.110
Follow-up (months, mean ± SD)	17.6 ± 6.8	18.5 ± 7.7	18.1 ± 6.4	0.915
Side (R/L)	15/12 (55.6/44.4%)	10/9 (52.6/47.4%)	10/8 (55.6/44.4%)	0.977
Fracture type: Transverse	15 (55.6%)	8 (42.1%)	9 (50.0%)	0.791
Fracture type: Oblique	11 (40.7%)	9 (47.4%)	7 (38.9%)	0.791
Fracture type: Comminuted	1 (3.7%)	2 (10.5%)	2 (11.1%)	0.791
Concomitant fibula fracture (Yes/No)	16/11 (59.3/40.7%)	14/5 (73.7/26.3%)	13/5 (72.2/27.8%)	0.512
Complications (Yes/No)	4/23 (14.8/85.2%)	3/16 (15.8/84.2%)	3/15 (16.7/83.3%)	0.986

Significant at *p* < 0.05 level, ESIN: Elastic Stable Intramedullary Nail.

**Table 2:** (a) Comparison of coronal plane alignment between groups at preoperative, postoperative, and final follow-up. (b) Comparison of sagittal plane alignment between groups at preoperative, postoperative, and final follow-up.

(**a**)				
	group			
	K wire	Plate	ESIN	
	Ort ± SS	Ort ± SS	Ort ± SS	*p*
Preoperative translation—Coronal (mm)	14.2 ± 3.56	13.92 ± 3.25	14.22 ± 3.64	0.141 ǂ
Postoperative translation—Coronal	1.97 ± 1	0.09 ± 0.1	1.7 ± 0.9	
Final translation (mm)	0 ± 0	0 ± 0	0 ± 0	
*p*	0.001 *†			0.229 ỻ
Preoperative angulation coronal—Varus (−), valgus (+)	14.8 ± 5.31	14.31 ± 4.5	13.75 ± 4.58	0.948 ǂ
Postoperative angulation coronal—Varus (−), valgus (+)	3.09 ± 0.93	0.82 ± 0.14	3.15 ± 0.74	
Final angulation—coronal varus valgus	0.47 ± 0.14	0.51 ± 0.12	0.55 ± 0.15	
*p*	0.010 *†			0.975 ỻ
(**b**)				
	group			
	K wire	Plate	ESIN	
	Ort ± SS	Ort ± SS	Ort ± SS	*p*
Preoperative translation—Sagittal	12.62 ± 2.07	12.78 ± 1.97	12.57 ± 2.13	0.191 ǂ
Postoperative translation—Sagittal	1.41 ± 0.62	0.08 ± 0.09	1.27 ± 0.47	
Final translation (mm)	0 ± 0	0 ± 0	0 ± 0	
*p*	0.001 *†			0.054 ỻ
Preoperative angulation—Sagittal procurvatum (−), recurvatum(+)	9.74 ± 3.69	11.75 ± 2.35	10.67 ± 2.98	0.884 ǂ
Postoperative angulation—Sagittal procurvatum (−), recurvatum (+)	1.21 ± 0.43	0.46 ± 0.09	1.03 ± 0.24	
Final sagittal—procurvatum (−), recurvatum (+)	0.18 ± 0.09	0.12 ± 0.08	0.13 ± 0.09	
*p*	0.001 *†			0.001 *ỻ

* Statistically significant at *p* < 0.05, two-way repeated measures ANOVA, † time, ǂ group, ỻ group × time interaction; ESIN: Elastic Stable Intramedullary Nail.

**Table 3:** Comparison of AOFAS scores between groups.

AOFAS	K Wire	Plate	ESIN	*p*
AOFAS (postoperative 4th month)	74.78 ± 5.23	85 ± 3.14	75.22 ± 5.83	0.001 *ǂ
AOFAS (postoperative 8th month)	89.22 ± 3.27	90.84 ± 2.57	89.94 ± 4.08	
AOFAS (postoperative final follow-up year)	99.07 ± 0.96	99.32 ± 0.82	99.11 ± 0.96	
*p*	0.001 *†			0.001 *ỻ

* Statistically significant at *p* < 0.05, two-way repeated measures ANOVA, † time, ǂ group, ỻ group × time interaction; AOFAS score: American Orthopedic Foot and Ankle Society score.

## Data Availability

The datasets used and/or analysed during the current study are available from the corresponding author on reasonable request.

## Abbreviations

The following abbreviations are used in this manuscript:

DDMJ	Distal diaphyseal–metaphyseal junction
ESIN	Elastic stable intramedullary nail
AOFAS	American Orthopedic Foot and Ankle Society
K-wire	Kirschner wire
